# Downstaging and survival after Neoadjuvant chemotherapy for bladder cancer in Norway; a population-based study

**DOI:** 10.1186/s12885-022-10394-w

**Published:** 2022-12-12

**Authors:** Christina Tanem Møller, Nathalie C. Støer, Augun Blindheim, Viktor Berge, Gunnar Tafjord, Sophie D. Fosså, Bettina Kulle Andreassen

**Affiliations:** 1 Department of Research, Cancer Registry of Norway, Pb 5313 Majorstuen, 0304 Oslo, Norway; 2 Faculty of Medicine, University of Oslo, Oslo, Norway; 3 Department of Clinical and Molecular Medicine, Norwegian University of Science and Technology (NTNU), Trondheim, Norway; 4 Department of Surgery, St. Olavs Hospital, Trondheim University Hospital, Trondheim, Norway; 5 Department of Urology, Oslo University Hospital, Oslo, Norway; 6 Department of Oncology, Oslo University Hospital, Oslo, Norway; 7 National Advisory Unit on Late Effects after Cancer Treatment, Oslo University Hospital, Oslo, Norway

**Keywords:** Bladder cancer, Neoadjuvant chemotherapy, Pathological downstaging, Population-based, Overall survival

## Abstract

**Background:**

Neoadjuvant chemotherapy (NAC) before radical cystectomy is associated with pathological downstaging (DS) and improved overall survival (OS) in patients with muscle-invasive bladder cancer (MIBC). Population-based studies have not unequivocally shown improved survival. The aim of this population-based study was to evaluate the effect of NAC on DS and OS in Norwegian patients with MIBC.

**Methods:**

Patients in the Cancer Registry of Norway undergoing radical cystectomy (2008–2015) with or without NAC diagnosed with MIBC between 2008 and 2012 were included. Follow-up data were available until 31 December 2019. Logistic regression estimated the odds of DS with NAC, and a Cox model investigated the effect of DS on OS. Cox models, a mediator analysis and an instrumental variable approach were used to investigate the effect of NAC on OS.

**Results:**

A total of 575 patients were included. NAC was administered to 82 (14%) patients. Compared to cystectomy only, NAC increased the proportion (43% vs. 22%) and the odds of DS (OR 2.51, CI 1.37–4.60, *p* = 0.003). Independent of NAC, the proportion of pN0 was higher in patients with DS (89% vs. 60%) and DS yielded a 78% mortality risk reduction (HR 0.22, CI 0.15–0.34, *p* = 1.9∙10–12), compared to patients without DS. We did not find an association between NAC and OS, neither by Cox regression (HR 1.16, CI 0.80–1.68, *p* = 0.417) nor by an instrumental variable approach (HR = 0.56, CI = 0.07–4.57, *p* = 0.586). The mediation analysis (*p* = 0.026) confirmed an indirect effect of NAC on OS through DS. Limitations include limited information of the primary tumour, details of NAC treatment and treatment indications.

**Conclusions:**

NAC increases the probability of DS and is indirectly associated to OS. DS is related to the absence of regional lymph node metastases and is associated with an OS benefit. Improved staging and biomarkers are needed to identify patients most likely to achieve DS and to benefit from NAC.

**Supplementary Information:**

The online version contains supplementary material available at 10.1186/s12885-022-10394-w.

## Background

In Europe [1] and in the USA [2], *cisplatin-containing neoadjuvant chemotherapy (NAC)* before radical cystectomy (RC) is recommended for patients with localized *(T2-T4a, cN0, M0)* muscle-invasive bladder cancer (MIBC) fit for cisplatin treatment. The European Association of Urology recommended NAC for MIBC in the 2008 guidelines [1] after several randomized controlled trials (RCT) [3–6] and meta-analyses [7, 8] had demonstrated a beneficial effect of NAC in MIBC. The survival benefit of NAC found in RCTs has been shown to be associated with *pathologic downstaging (DS*) of the primary tumour in the RC-specimen [6, 9, 10].

Meta-analyses based on results from RCTs have shown an absolute five-year *overall survival (OS)* benefit of 6–8% favouring NAC over RC alone [7, 8, 11]. Results from population-based studies have been inconclusive. Some authors did not find an association between NAC and improved survival [12, 13], while others did show a survival benefit for NAC relative to RC alone [14]. With this background, more data and analyses are warranted to establish the beneficial effect of NAC on a population-based level. Therefore, we aimed to describe the clinical characteristics of an unselected population of Norwegian patients with MIBC treated with NAC and RC (*NAC group*) vs. RC only (*NoNAC group*) and to evaluate the association between NAC and DS, the effect of DS on OS and the overall association between NAC and OS.

## Methods

### Material

*The Cancer Registry of Norway (CRN)* captures nearly 99% of new cancer diagnoses in Norway [15]. The collected data includes patient demographics, tumour characteristics, treatment codes (surgical, radiotherapy) and causes of death. For bladder cancer, histopathology of specimens from transurethral resection of bladder tumour (TURB), cystectomy and biopsies of metastases are registered, along with the corresponding dates for the procedures.

*The Norwegian Patient Registry* contains individual administrative, demographic, and coded medical information (diagnoses, procedures, chemotherapy) from all patients’ contacts with public hospitals. This data was linked to the CRN by the personal identification number assigned to all residents of Norway [16].

### Study population

We included patients undergoing RC (2008–2015) with or without NAC who were diagnosed with MIBC (*urothelial carcinoma)* without known distant metastases between 2008 and 2012. The pre-RC status of regional lymph node metastases was unknown (cNx). Patients with a pre-RC histology verifying muscle-invasion and patients without such verification but treated with NAC were considered as having MIBC. We chose this period since we had quality ensured histopathological information from this period and to ensure sufficient follow-up time for survival analysis. Patients with pre-RC radiotherapy were excluded.

### Measures

#### Muscle invasion

For the evaluable patients, the research team reviewed all available clinical notifications and histology reports at the CRN. The presence of MIBC was confirmed in the histology reports from TURB specimens. Muscle-invasion was defined as tumour invasion into the muscularis propria (≥T2). From the histology reports from cystectomy specimens, the histopathological T and N category (pT; pN) [17] without sub-classification into a and b for pT2-pT4 were confirmed. All MIBC are high-grade [18].

#### Neoadjuvant chemotherapy

We identified relevant specified intravenous chemotherapy codes (e.g., gemcitabine and cisplatin, methotrexate, vinblastine, doxorubicin and cisplatin (MVAC), carboplatin) and codes for intravenous administration of non-specified chemotherapy from the Norwegian Patient Registry. We excluded chemotherapy events concurrently registered with ICD-10 codes for a different cancer than C67. NAC was defined as any chemotherapy administered intravenously between diagnosis of bladder cancer and RC.

#### Downstaging

Based on the available data and definitions used in similar studies [12, 14, 19], we defined *downstaging of the primary tumour (DS)* as pT0/pTa/pTis/pT1 with the subunit of pT0 as *complete response (CR)*, identified independent of the use of NAC. Patients without DS *(non-DS)* were characterized by having residual muscle-invasive disease *(*pT2-pT4) in the specimen*.* Downstaging can occur after TURB and NAC. Nodal downstaging could not be assessed because information about cN was not available.

### Statistical analyses

The observation time started at the date of RC until death, emigration, or end of study (December 31st, 2019), whichever came first. Time in years from date of RC was used as timescale in all analyses.

We applied descriptive statistics (mean, median, interquartile range (IQR), proportions) to present pre- and post-operative characteristics in all patients as well as in the NAC and NoNAC group. The association between NAC and DS was estimated using logistic regression adjusted for all available *pre-operative variables*: age at diagnosis (≤59, 60–69, 70–79, ≥80), sex, type of hospital *(academic* vs. *community)*, geographical health region *(Southeast, West, Central, North)* and the year of RC *(2008–2009, 2010–2011, 2012–2015)*. OS was presented by Kaplan-Meier curves and the difference between them was evaluated with the log-rank test. The associations between DS with OS, as well as NAC with OS (*total effect*) were assessed with a Cox regression model adjusted for all available pre-operative variables.

The association between NAC and OS was additionally investigated by applying a mediation approach adjusted for available pre-operative variables. We applied a causal inference approach [20, 21] implemented in the R mediation package [22]. The *total* effect of NAC on OS (unadjusted for DS) evaluated with a Cox regression model was decomposed into two parts [23]: the *indirect* effect between NAC and OS mediated by DS, and the *direct* effect between NAC and OS (not through DS) This approach allowed us to assess the indirect effect of NAC on OS through DS.

In order to overcome the confounding by indication bias induced by missing information of factors leading to the decision of treatment, we applied an instrumental variable approach to estimate the causal effect of NAC on OS [24]. We used type of hospital as the instrumental variable and G-estimation [24–26] with adjustment for the remaining pre-operative variables.

Quantities reported from the model-based analyses are odds ratios (ORs) and hazard ratios (HRs) including 95% confidence intervals (CI) and *p*-values. The statistical significance level was set at 0.05. Statistical analyses were performed using Stata 17 (StataCorp, College Station, TX) and R (version 4.1.4).

## Results

### Patient characteristics

Between 2008 and 12, 5521 patients were diagnosed with first-time diagnosis of bladder cancer (urothelial carcinoma) and 917 of these patients underwent RC by the end of 2015. After exclusions, 575 patients were finally evaluable in our study (Supplementary Fig. S1): 82 (14%) patients in the NAC group and 493 (86%) patients in the NoNAC group. In the NAC group, 23 (28%) patients received gemcitabine and cisplatin, 10 (12%) patients MVAC and 1 (1%) patient Carboplatin. For 48 (59%) patients, the type of chemotherapy was unknown. The median follow-up time was 3.9 years.

The median age at diagnosis for the evaluable patients was 69 (IQR: 62,75) years and 124 (22%) of the patients were female (Table 1). Patients in the NAC group were younger (median 65 vs. 70 years), more frequently female (29% vs 20%) and more likely operated in an academic hospital (76% vs 61%) compared to the NoNAC group. Median time from the most recent TURB to cystectomy was 48 days for the patients undergoing cystectomy only, and 109 days for patients treated with NAC. The proportion of patients treated with NAC was increasing over time, with the largest proportions of patients (70%) treated between 2012 and 2015. Among the 82 patients in the NAC group, 47 (57%) patients died, compared to 301 (61%) patients out of 493 patients in the NoNAC group. The proportion of deaths due to other causes was larger in the NoNAC group (20% vs 12%) compared to the NAC group.

**Table 1:** Patient characteristics of the study population with respect to treatment

	NAC^**a**^	NoNAC^**b**^	All
**Patients, n (%)**	**82 (14%)**	**493 (86%)**	**575 (100%)**
Age, median (IQR)	65 (56,68)	70 (63,76)	69 (62,75)
Females, n (%)	24 (29%)	100 (20%)	124 (22%)
Academic hospital, n (%)	62 (76%)	299 (61%)	361 (63%)
Health region, n (%)			
Southeast	40 (49%)	274 (56%)	314 (55%)
West	22 (27%)	99 (20%)	121 (21%)
Central	11 (13%)	57 (12%)	68 (12%)
North	9 (11%)	63 (13%)	72 (13%)
Cystectomy year, n (%)			
2008–2009	16 (20%)	172 (35%)	188 (33%)
2010–2011	9 (11%)	211 (43%)	220 (38%)
2012–2015	57 (70%)	110 (22%)	167 (29%)
Number of deaths, n (%)	47 (57%)	301 (61%)	348 (60%)
Cause of death, n (%)			
Bladder cancer	37 (45%)	204 (41%)	241 (42%)
Other causes	10 (12%)	97 (20%)	107 (19%)

^a^ *NAC* Pre-cystectomy neoadjuvant chemotherapy

^b^ *NoNAC* Cystectomy only

Out of 575 patients, pT was recorded in 514 (89%) patients and thus evaluable for DS, and pN was recorded for 433 (75%) of patients (Table 2). The proportions of pT3 (47% vs 27%) and pN+ (35% vs 25%) in the NoNAC group were larger compared to the NAC group, while the proportion of CR (9% vs 24%) was smaller. Out of 29 patients with DS in the NAC group, 16 (55%) patients had CR, whilst 38 (40%) out of 96 patients with DS in the NoNAC group had CR.

**Table 2:** Postoperative tumour characteristics for patients with available histopathological information according to treatment

	NAC^**a**^	NoNAC^**b**^	All
**Patients, n (%)**	**67 (13%)**	**447 (87%)**	**514 (100%)**
Pathological T category, n (%)			
*Downstaging of primary tumour*			
pT0	16 (24%)	38 (9%)	54 (11%)
pTa	2 (3%)	13(3%)	15 (3%)
pTis	6 (9%)	21(5%)	27 (5%)
pT1	5 (7%)	24(5%)	29 (6%)
*Residual muscle-invasive disease*			
pT2	12 (18%)	92 (21%)	104 (20%)
pT3	18 (27%)	212 (47%)	230 (45%)
pT4	8 (12%)	47 (11%)	55 (11%)
Pathological N category, n (%)	**65 (15%)**	**368 (85%)**	**433 (100%)**
pN+	16 (25%)	129 (35%)	145 (33%)
pN0	49 (75%)	239 (65%)	288 (67%)

^a^ *NAC*: Pre-cystectomy neoadjuvant chemotherapy

^b^ *NoNAC* Cystectomy only

Out of 514 patients evaluable for DS, pN was recorded for 427(83%) patients (Supplementary table S1). The proportion of patients with pN0 among patients with DS (89% vs 60%) was larger compared to patients with non-DS without difference between patients treated with and without NAC (92% vs 88%).

### Neoadjuvant chemotherapy and downstaging

**Fig. 1:**
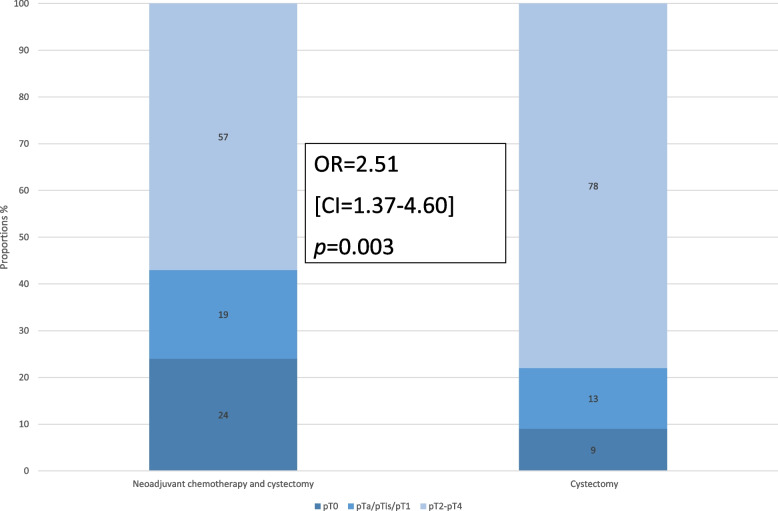
Comparison of the proportions of patients with (pT0/pTa/pTis/pT1) and without downstaging (pT2-pT4) by treatment. Logistic regression results (odds ratio OR, 95% confidence interval CI and *p*-value)

Compared to patients in the NoNAC group, a larger proportion of patients achieved DS (43% vs. 22%) in the NAC group (Fig. 1). NAC significantly increased the odds for DS (OR 2.51, CI 1.37–4.60, *p* = 0.003) compared to NoNAC (Supplementary table S2).

### Downstaging and overall survival

**Fig. 2:**
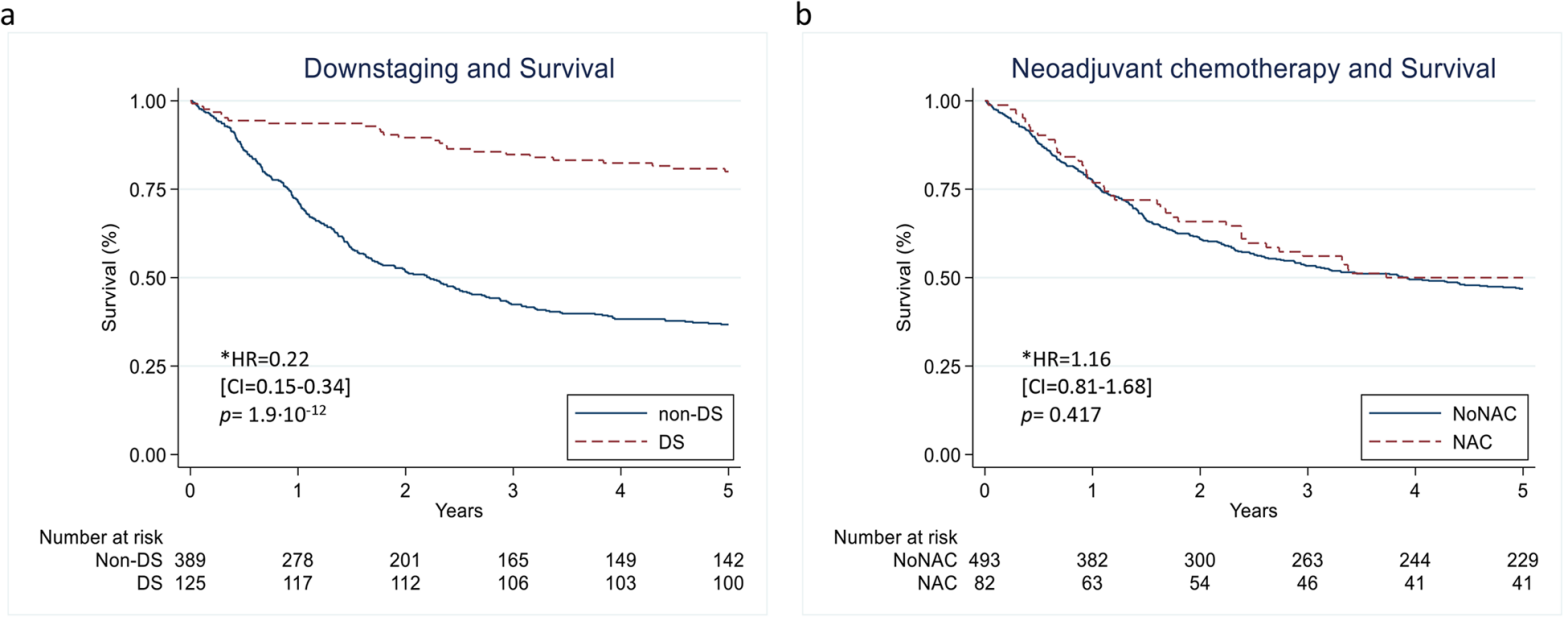
Kaplan-Meier curves of overall survival (OS). **a** OS in patients with downstaging (DS: ≤pT1) compared to patients without (non-DS) (*n* = 514) **b** OS in patients treated with neoadjuvant chemotherapy (NAC) compared to patients treated with cystectomy only (NoNAC) (*n* = 575); Cox regression results (hazard ratio HR, 95% confidence intervals CI and *p*-value)

For patients with DS, the crude five-year OS was larger compared to patients with non-DS (80% vs. 38%, *p* < 0.001) (Fig. 2 a). The adjusted survival analysis revealed a 78% risk reduction of all-cause death (HR 0.22, CI 0.15–0.34, *p* = 1.9∙10^− 12^) in patients with DS compared to patients with non-DS (Supplementary table S2).

### Neoadjuvant chemotherapy and overall survival

The crude five-year OS for all patients (*n* = 575) was 47%: NAC 50% vs. NoNAC 47% (Fig. 2 b). NAC was not significantly associated with OS in the crude analysis (*p* = 0.552), in the Cox analysis (HR 1.16, CI 0.80–1.68, *p* = 0.417) nor when we applied the instrumental variable approach (HR 0.56, CI 0.07–4.57, *p* = 0.586) (Supplementary table S3).

**Fig. 3:**
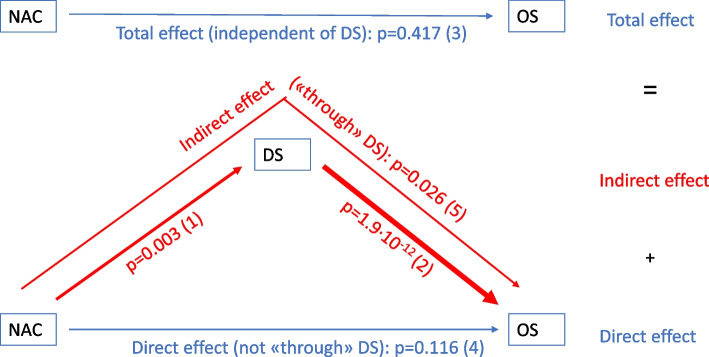
Summary of results. Summary of the results; logistic regression (1), Cox regression (2-3) and mediation analysis (4–5). (1) Neoadjuvant chemotherapy (NAC) is significantly associated with pathological downstaging (DS: ≤pT1), (2) DS is significantly associated with overall survival (OS), (3) NAC is not significantly associated with OS (unadjusted for DS*) (Total effect)*, (4) NAC is not significantly associated with OS (not through DS) *(Direct effect),* (5) NAC is significantly associated with OS through DS *(Indirect effect)* (red arrow: significant; the thicker, the more significant; blue arrow: not significant). *All analyses were adjusted for age, sex, type of hospital, health region and cystectomy year

The mediation analysis confirmed the above results by revealing an indirect effect of NAC on OS through DS (*p* = 0.026), but no total or direct effect of NAC on OS (Fig. 3, Supplementary table S4).

## Discussion

In this population-based study, NAC increased the probability of achieving DS in patients with MIBC by a factor of 2.5. Independent of the means for obtaining DS (NAC or TURB), achievement of DS in MIBC patients was associated with a 78% risk reduction of all-cause mortality compared to non-DS and related to a decreased proportion of patients with regional node lymph node metastases verified in the RC specimen. NAC did not provide a beneficial survival over NoNAC, except for when the effect of NAC on OS went through DS.

Post-NAC DS was found in 43% of the patients in our study. In comparison, post-NAC DS was reported in 36% of patients in a US population-based study [14], in 51% of the patients in a large single-institution registry study [27] and in 61% in a Danish population-based study [19]. In two Nordic RCTs post-NAC DS was reported in 38% of the patients receiving cisplatin+doxyrubicin/methotrexate [9]. For the more modern chemotherapeutic regimens, the proportion of post-NAC DS was higher (Gemcitabine and cisplatin: 49%, dose dense MVAC: 63%) [28]. Different study designs have used different definition of DS. In our and other relevant population-based studies [12, 14, 19], DS was defined as downstaging of the primary tumour(<pT2) and independent of pN-status [12, 14, 19], whereas in selected clinical trials pN0 was included in the definition (<pT2pN0) [9, 28]. Notably DS can be the effect of NAC but can also be achieved after an extensive TURB.

We show that the proportions of pN0 was higher in patients with DS compared to patients with non-DS, although without any difference in downstaged patients treated with or without NAC. These results are in line with the corresponding combined results from two previous clinical trials [9]. Further, the demonstration of DS independent of the receipt of NAC revealed a beneficial survival, as patients with DS had a 78% risk reduction of all-cause death compared to patients without DS. These results indicate that independent of NAC, DS is related to the absence of regional lymph node metastases and indicates a more favourable prognosis compared to patients without DS. However, NAC significantly increased the odds of DS and possibly reflect the favourable effect of NAC on regional lymph node metastases and micrometastases.

Our findings of no survival benefit in the NAC group vs. NoNAC group is in agreement with the results from two other population-based studies from the US [12] and Sweden [13]. Despite efforts to account for selection bias and unrecognized confounders with statistical methods like propensity score weighting or the instrumental variable approach in our study, no OS benefit for NAC over NoNAC was found. However, we are the first to identify an indirect effect of NAC on survival through the demonstration of DS as we show that NAC has an effect on OS mediated by DS. We suggest the following explanations: The patients in the NAC group initially may have had a more advanced and aggressive disease compared to the patients in the NoNAC group, reducing the potential survival advantage gained by post-NAC DS when evaluating the total effect of NAC on OS. On the other hand, the population may consist of subgroups of patients who do not benefit from NAC, as the selection of patients treated with NAC in the real-world is most probably different from clinical trials [29]. Notably, in other population-based studies the proportions of cT2N0M0 (82–86%) [12, 13] were larger than in RCTs (34–40%) [5, 6, 30]. For this subgroup, RCTs have either not evaluated the mortality risk after NAC [5, 6] or found no survival benefit from NAC [30], and in two population-based studies no survival benefit over NoNAC was found [31, 32].

Our findings underline the necessity to determine which MIBC patients benefit from NAC in clinical practice. Identification of subgroups of patients most likely to achieve DS with or without NAC is necessary. The latter are of particular interest as they are possible candidates for bladder preserving strategies. Clinical staging by computed tomography is challenging with an estimated accuracy of 40–92% to predict pT and of 54–86% to predict pN [33]. Advances in image-guided approaches with multiparametric magnetic resonance imaging may reduce staging errors in the management of MIBC and aid in predicting treatment response to NAC [34]. Reliable biomarkers for chemotherapy sensitivity are needed.

Limitations of our study include the lack of pre-RC information about cT- and cN category and limited information about the primary tumour (lack of size, multiplicity, and widespread carcinoma in situ). To our knowledge, only cisplatin-based NAC was used in Norway in the study period. Although application details of NAC were not available to us, the results reflect the real-world situation where dosage reduction and uncompleted cycles often are necessary. We do not know *why* some patients received NAC and others did not (confounding by indication). For this reason, we applied the instrumental variable approach, although limited by a suboptimal instrumental variable and limited power. However, we had solid information on pT, and we were the first population-based study to apply a mediator analysis and identify an indirect effect of NAC on survival through DS.

## Conclusion

In this nationwide population-based study of patients with MIBC, we found that on a population-based level DS demonstrated in the RC specimen is a good prognostic factor and provides a survival benefit over non-DS. NAC increases the odds of DS and is indirectly associated with an OS benefit. DS is related to absence of regional lymph nodes. Future perspectives include improvement of clinical staging, identification of patient subgroups most likely to achieve DS or non-DS, and identification of patients in whom NAC is necessary to achieve DS.

## Supplementary Information

**Additional file 1: Figure S1.** Study population.

**Additional file 2: Table S1.** Pathological N-category after cystectomy with regards to downstaging of primary tumour and treatment.

## Data Availability

The data that support the findings of this study are available from the Cancer Registry of Norway, but restrictions apply to the availability of these data, which were used under license for the current study, and so are not publicly available. Data are however available from the authors upon reasonable request and with permission of the Cancer Registry of Norway.

## Abbreviations

CI: Confidence interval
CRN: Cancer Registry of Norway
DS: Pathological downstaging of the primary tumour
HR: Hazard ratio
IQR: Interquartile range
NAC: Neoadjuvant chemotherapy
Non-DS: Residual muscle-invasive bladder cancer
MIBC: Muscle-invasive bladder cancer
MVAC: Methotrexate, vinblastine, doxorubicin and cisplatin
OR: Odds ratio
OS: Overall survival
RC: Radical cystectomy
RCT: Randomized controlled trial
TURB: Transurethral resection of bladder tumour
