# Supplementary figures and images for: Downstaging and survival after Neoadjuvant chemotherapy for bladder cancer in Norway; a population-based study
Source: BMC Cancer. 2022 Dec 12;22:1301. doi: 10.1186/s12885-022-10394-w (PMC9746207; doi:10.1186/s12885-022-10394-w)

**Figure S1** Study population

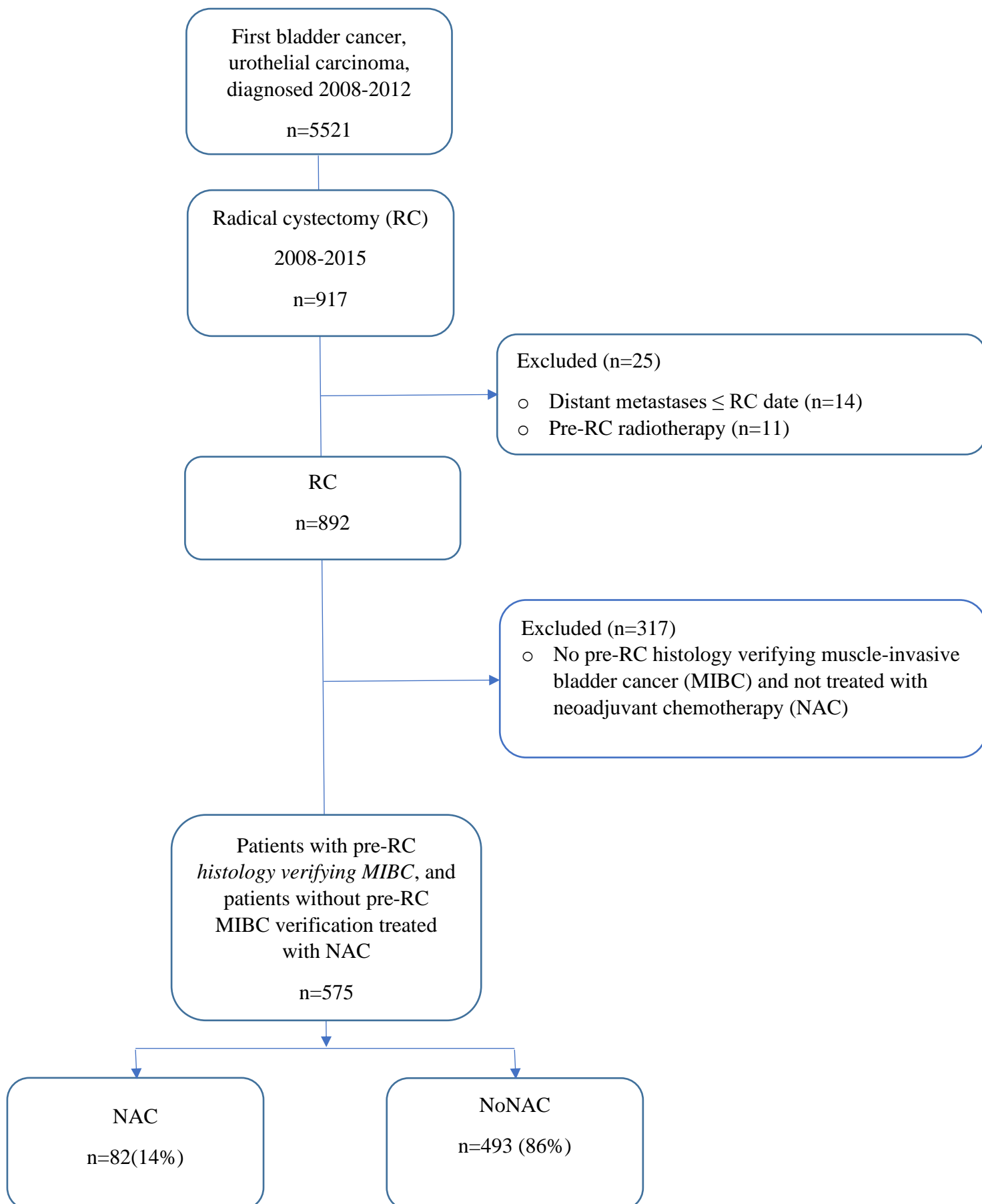

Supplement: Supplementary file 1 — Additional file 1: Figure S1. Study population. [file 12885_2022_10394_MOESM1_ESM.pdf]
