# Supplementary material for: Downstaging and survival after Neoadjuvant chemotherapy for bladder cancer in Norway; a population-based study
Source: BMC Cancer. 2022 Dec 12;22:1301. doi: 10.1186/s12885-022-10394-w (PMC9746207; doi:10.1186/s12885-022-10394-w)
Supplement: Supplementary file 2 — Additional file 2: Table S1. Pathological N-category after cystectomy with regards to downstaging of primary tumour and treatment. [file 12885_2022_10394_MOESM2_ESM.pdf]

Supplementary

Table S1 Pathological N-category after cystectomy with regards to downstaging of primary tumour and treatment

	**Neoadjuvant chemotherapy**			**Cystectomy only**			**Total**		
	**pN0**	**pN+**	**Total**	**pN0**	**pN+**	**Total**	**pN0**	**pN+**	**Total**
**Downstaging (<pT2), n (%)**	24(92%)	2(8%)	26	67(88%)	9(12%)	76	91(89%)	11(11%)	102
pT0	14(93%)	1(7%)	15	29(91%)	3(9%)	32	43(91%)	4(9%)	47
pTa, pTis, pT1	10(91%)	1(9%)	11	38(86%)	6(14%)	44	48(87%)	7(13%)	55
**Residual muscle-invasive disease (≥pT2), n (%)**	23(64%)	13(36%)	36	171(59%)	118(41%)	289	194(60%)	131(40%)	325

Table S2 Association between neoadjuvant chemotherapy and downstaging and downstaging and overall survival (n=514)

		Downstaging[a*]			Overall survival[b*]		
		**OR**	**CI**	***p* value**	**HR**	**CI**	***p* value**
**Neoadjuvant chemotherapy**	No	1					
	Yes	**2.51**	**1.37-4.60**	**0.003**			
**Downstaging (<pT2)**	No				**1**		
	Yes				**0.22**	**0.15-0.34**	**$1.9 \cdot 10^{-12}$**
**Age**	≤59	1			1		
	60-69	0.95	0.53-1.69	0.858	1.14	0.77-1.69	0.497
	70-79	0.58	0.32-1.07	0.085	1.19	0.81-1.75	0.386
	≥80	0.16	0.05-0.59	**0.005**	1.42	0.87-2.30	0.162
**Sex**	Male	1			1		
	Female	0.71	0.41-1.23	0.222	1.03	0.77-1.39	0.817
**Academic hospital**	No	1			1		
	Yes	0.79	0.46-1.33	0.367	0.83	0.62-1.11	0.208
**Health region**	Southeast	1			1		
	West	1.10	0.58-2.06	0.775	0.99	0.68-1.42	0.941
	Central	1.35	0.67-2.72	0.403	0.96	0.63-1.48	0.867
	North	0.91	0.47-1.77	0.790	1.35	0.94-1.93	0.102
**Year of cystectomy**	2008-2009	1			1		
	2010-2011	0.95	0.57-1.56	0.828	1.22	0.91-1.63	0.178
	2012-2015	0.79	0.45-1.39	0.422	1.08	0.79-1.48	0.611

[a] **Logistic regression; odds ratio OR, 95% confidence intervals CI and *p*-values**

[b] **Cox regression; hazard ratio HR, 95% confidence intervals CI and *p*-values**

*** Adjusted for Age, Sex, Academic Hospital, Health Region and the Year of Cystectomy**

Table S3 The impact of neoadjuvant chemotherapy on overall survival for all patients (n=575)

		**Overall survival[a]**		
		**HR**	**CI**	***p* value**
**Neoadjuvant chemotherapy**	No	1		
	Yes	**1.16**	**0.81-1.68**	**0.417**
**Age**	≤59	1		
	60-69	1.25	0.87-1.79	0.237
	70-79	1.44	1.0-2.10	0.048
	≥80	2.07	1.32-3.27	0.002
**Sex**	Male	1		
	Female	1.04	0.79-1.37	0.762
**Academic hospital**	No	1		
	Yes	0.90	0.69-1.20	0.490
**Health region**	Southeast	1		
	West	0.91	0.65-1.29	0.592
	Central	0.89	0.59-1-35	0.583
	North	1.18	0.84-1.67	0.346
**Year of cystectomy**	2008-2009	1		
	2010-2011	1.26	0.96-1.65	0.100
	2012-2015	0.95	0.70-1.29	0.745
		**Overall survival[b]**		
		**HR**	**CI**	***p* value**
**Neoadjuvant chemotherapy**	No	1		
	Yes	**0.56**	**0.07-4.57**	**p=0.586**

[a] Cox regression; hazard ratio HR, 95% confidence intervals CI and *p*-values

[b] Instrumental variable; hazard ratio HR, 95% confidence intervals CI and *p*-values

Table S4 Results from the mediation analysis* of neoadjuvant chemotherapy (NAC) on overall survival (OS)**

	**Effect estimate**	**95% confidence interval**	**p value**
**Direct effect of NAC on OS, adjusted for DS**	-2076.5	-4481.9 - 486.2	p=0.116
**Indirect effect of NAC on OS through DS**	1550.8	227.1 - 3625.5	p=0.026

*Mediation variable downstaging (DS: <pT2)

**Adjusted for age, sex, health region, type of hospital, year of cystectomy
